# The effect of low-dose esketamine on pain and post-partum depression after cesarean section: A prospective, randomized, double-blind clinical trial

**DOI:** 10.3389/fpsyt.2022.1038379

**Published:** 2023-01-04

**Authors:** Jiahong Shen, Congzhong Song, Xinlei Lu, Yuxin Wen, Shaobo Song, Jing Yu, Jianliang Sun

**Affiliations:** ^1^Department of Anesthesiology, Affiliated Hangzhou First People’s Hospital, Zhejiang University School of Medicine, Hangzhou, China; ^2^Department of Anesthesiology, Affiliated Xiaoshan Hospital, Hangzhou Normal University (Zhejiang Xiaoshan Hospital), Hangzhou, China

**Keywords:** esketamine, pain, post-partum depression, prophylactic, cesarean delivery

## Abstract

**Objective:**

To observe and evaluate the effect of a single intravenous injection of low-dose esketamine on post-operative pain and post-partum depression (PPD) in cesarean delivery patients.

**Methods:**

A total of 210 patients undergoing elective cesarean delivery under combined spinal-epidural anesthesia were divided into an esketamine group (Group S, *n* = 105) and a normal saline group (Group L, *n* = 105) by a random number table. At 5 min after childbirth, patients in the S group were given 0.25 mg/kg esketamine, whereas patients in the L group received an equal volume of saline. The primary outcomes included post-operative pain control according to the Numerical Rating Scale (NRS) and the incidence of PPD according to the Edinburgh Post-partum Depression Scale (EPDS). The secondary outcomes included analgesia-related adverse events and Ramsay sedation scores.

**Results:**

This clinical study was a prospective, randomized, double-blind trial. A total of 210 patients were enrolled in this study. The NRS pain (cough pain) score was lower in the S group than in the L group at 24 h after surgery (*P* = 0.016), and there was no significant difference in resting pain and mobilization pain at 4, 8, and 48 h after surgery or resting pain at 24 h after surgery between the two groups. There was no significant difference in the prevalence of PPD between the two groups on the day before delivery, or at the first week, the second week, or the fourth week after childbirth. At 5 min after dosing, the incidence of hallucinations (*P* < 0.001) and dizziness (*P* < 0.001) was higher in the S group than in the L group. At 15 min after dosing, the incidence of dizziness (*P* < 0.001) and nausea (*P* = 0.011) was higher in the S group than in the L group. The incidence of dizziness (*P* < 0.001) was higher in the S group than in the L group when leaving the operating room. The Ramsay scores in Group S were lower than in Group L at 5 min (*p* < 0.001), 15 min (*p* < 0.001) after dosing and at the time of leaving the operating room (*p* < 0.001).

**Conclusion:**

In this study, a single intravenous injection of 0.25 mg/kg esketamine did not reduce the incidence of depression at 1, 2, or 4 w post-partum but improved pain during exercise at 24 h post-operatively under the conditions of this clinical trial.

**Clinical trial registration:**

[www.chictr.org.cn], identifier [ChiCTR2100054332].

## Introduction

Post-partum depression (PPD) is one of the most common psychiatric disorders in the perinatal period and has become an important public health problem, yet it remains underdiagnosed and undertreated to date. The prevalence of PPD varies in different countries, ranging from 6.9 to 12.9% in economically developed countries, while it is as high as more than 20% in developing or poor countries (1). A meta-analysis by Gavin et al. showed that the incidence of depression during pregnancy was 18.4%, the incidence of major depression was 12.7%, the incidence of depression within 3 months after delivery was 19.2%, and the incidence of major depression was 7.1% (2). Norhayati (3) reviewed 191 studies from 42 countries using self-questionnaire surveys from 2005 to 2014 and found that the prevalence of PPD ranged from 1.9 to 82.1% in developing countries and from 5.2 to 74.0% in developed countries. In conclusion, although the incidence of PPD reported by different investigators varies greatly, PPD is indeed a ubiquitous perinatal mental disorder, which should be given enough attention by the majority of medical workers.

The American College of Obstetricians and Gynecologists (4), the American Academy of Pediatrics (5), and the US Preventive Services Task Force (6) recommend routine post-partum emotional testing and the assessment of post-partum women using the 10-item Edinburgh Post-partum Depression Scale (EPDS) to maximize early screening and confirmation of PPD. At the same time, depression and anxiety symptoms caused by thyroid dysfunction and anemia (7), which are common in post-partum women, should be excluded.

There are many pathogenic factors of PPD, such as trauma stimulation, endocrine changes, psychological stress, and social factors (8). Moreover, studies have shown that pain is strongly correlated with depression (9), and any pain will lead to some behavioral and psycho-physical effects.

Esketamine, the S enantiomer of ketamine, is an NMDA receptor antagonist that exerts an antidepressant effect by blocking NMDA receptors and activating AMPA (α-amino-3-hydroxy-5-methyl-4 isoxazolpropionic acid) receptors (10). It can also act on neurotransmitter systems, such as the cholinergic, γ-aminobutyric acid, monoaminergic and opioid systems, and has significant analgesic effects (11). Therefore, esketamine is a sedative, an analgesic, and an antidepressant. Therefore, according to the rapid antidepressant and analgesic effects of esketamine, we propose the following scientific hypothesis: the application of esketamine during cesarean section can effectively reduce pain after cesarean section and is expected to reduce the incidence of PPD in women who undergo cesarean section, which has important social significance.

However, whether esketamine can reduce the incidence of PPD is still lacking clinical evidence. Therefore, we designed this prospective, randomized, double-blind trial to evaluate the effect of a single low-dose intravenous infusion of esketamine (0.25 mg/kg) during cesarean section on post-operative pain and PPD.

## Materials and methods

### Research object

This study was a randomized controlled double-blind trial. We obtained approval from Zhejiang Xiaoshan Hospital. Written informed consent was obtained from all pregnant women. This trial was registered with the Chinese Clinical Trial Registry (registration number ChiCTR2100054332).

The selection criteria were as follows: (1) pregnant women at 36∼42 w of gestation with a singleton pregnancy and scheduled cesarean delivery; (2) those with ASA scores of I-II; (3) those aged 18–40 years; (4) those who had a weight of less than 90 kg and a height of at least 150 cm; (5) those with good communication with their partner; and (6) those who signed the informed consent form.

The exclusion criteria were as follows: (1) an allergy to the drugs used in this study; (2) long-term use of narcotic analgesics, sedatives, or non-steroidal anti-inflammatory drugs; (3) unstable psychiatric disorders; (4) severe electrocardiogram abnormalities; (5) severe hypertension and severe heart diseases; (6) drug or alcohol dependence for more than 6 months; (7) a prior ineffective trial of or adverse reaction to esketamine; and (8) refusal to provide written informed consent.

### Research design

According to the coding sequence, 210 parturients were randomly divided into the esketamine group (Group S, *n* = 105) and saline group (Group L, *n* = 105). At 5 min after neonatal delivery, 0.25 mg/kg esketamine (normal saline diluted to 10 ml) was injected intravenously in Group S, and the same amount of normal saline was injected intravenously in Group L.

None of the women used pre-operative medication. After the upper limb venous infusion channel was established by operating room nurses, anesthesiologists connected the corresponding vital sign monitoring systems, including ECG, blood pressure, and pulse oxygen saturation (SpO2) monitoring systems, for three consecutive measurements of the mean arterial pressure (MAP) and an average value basis for the MAP. All puerperae were placed in the left lateral decubitus position. After routine skin disinfection and 3–5 ml local infiltration anesthesia with lidocaine, an epidural puncture was performed with an 18 Tuohy needle in the L3-4 intervertebral disc space using the resistance disappear method. A 26 lumbar hemp pencil type needle set was successfully inserted into the lumbar hemp Tuohy needle line puncture, with the pinhole direction toward the head end, until the cerebrospinal fluid flow was clear. Within 10∼20 s, 15 mg of a 0.5% ropivacaine mixture (2 ml of 0.75% ropivacaine injection + normal saline to the total volume of 3 ml) was injected. After the infusion, the lumbar anesthesia needle was withdrawn, and a 2∼3 cm epidural tube was placed into the head. After the completion of anesthesia, the mother was placed in the supine position, and a 15-degree wedge was placed on the right hip to prevent supine hypotension syndrome. The block plane was measured over time. If the block plane did not reach the T6 level, 5 ml of 2% lidocaine was added to the epidural every 5 min until the block plane reached the T6 level. Cesarean section was performed when the bilateral sensory block plane block reached the T6 level or above. Maternal vital signs were closely monitored, and 6 μg of norepinephrine was administered intravenously (reusable) if maternal hypotension occurred (MAP ≤ 80% of the MAP base value or an MAP < 60 mmHg) occurred, and 6 mg of ephedrine was administered intravenously (reusable) if bradycardia occurred (ventricular rate < 50 BPM).

In the experimental group, 0.25 mg/kg esketamine was diluted to 10 ml in normal saline and injected slowly, while in the control group, 10 ml saline was injected slowly. The anesthesiologist responsible for the implementation of anesthesia did not know about the medication assignment. 10 min before the end of the operation, 0.4 mg hydromorphone (diluted to 5 ml in normal saline) was administered in the epidural space for post-operative analgesia, and the epidural catheter was removed after the operation.

After the operation, a patient-controlled intravenous analgesia (PCIA) pump was administered, with Butorphanol (50 μg/ml) + tramadol (3 mg/ml) + Ondansetron (80 μg/ml), a total volume of 100 ml, a background infusion of 1 ml/h, a self-controlled infusion of 3 ml/time, a locking time of 10 min, a maximum dose of 12 ml/h, and an analgesia duration of 48 h after the operation. If the analgesic drugs were used up within 48 h, the dosage was refilled.

The patients were followed up by another trained investigator blinded to the experimental procedure in the hospital who recorded Numerical Rating Scale (NRS) scores at 4, 8, 24, and 48 h at rest and during exercise (i.e., when coughing). The EPDS scores of the patients were investigated by a bedside questionnaire 1 day before delivery and telephone interviews 1, 2, and 4 weeks after delivery.

### Outcomes

All patients were followed up for 4 weeks and those who were lost to follow-up were excluded from this study. The primary outcomes included post-operative pain control and the incidence of PPD. Post-operative pain control was assessed by NRS, which ranged from 0 (painless) to 10 (most severely painful). PPD was assessed by EPDS. The EPDS has 10 questions, with a total score of 30 points. An EPDS score of nine or more indicated the existence of PPD. The evaluation of the EPDS scale and the diagnosis of PPD were completed by Psychiatrists. The secondary outcomes included analgesia-related adverse events (dizziness, hallucinations, nausea, headache, vomiting, diplopia, and blurred vision) and Ramsay sedation scores.

Ramsay Sedation Scale: 1 = patient anxiety and restlessness; 2 = patient cooperation, orientation, quiet; 3 = patient responds to instructions; 4 = lethargic, responsive to tapping between the eyebrows or loud auditory stimuli; 5 = lethargic, sluggish response to tapping between the eyebrows or loud auditory stimuli; 6 = lethargic, no response.

### Statistical analyses

All of the individual participant data were analysed using SPSS version 26.0 (IBM SPSS Inc., Armonk, United States). Descriptive statistics were obtained for all the study variables. Data are reported as the means (standard deviations), medians (range), or frequencies (percentages) where appropriate. The Shapiro-Wilk test was applied to determine whether continuous variables were normally distributed. Independent sample *t* test analysis was adopted for the comparison of normally distributed quantitative data. Non-normally distributed data were analysed using the Mann–Whitney *U* test. Categorical variables were compared using the χ^2^ test or Fisher’s exact test, where appropriate. The *p* value was set lower than 0.05 for statistical significance.

## Results

**FIGURE 1:**
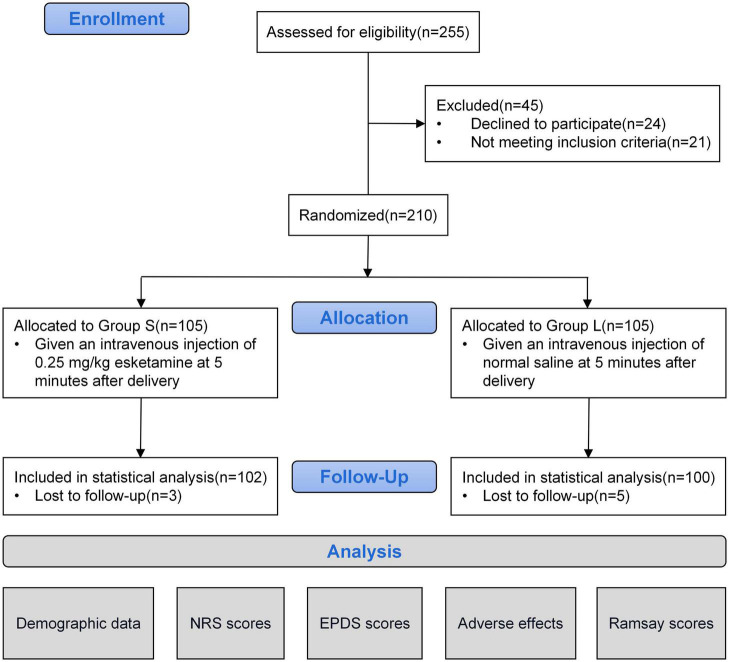
Flow chart.

A total of 255 parturients were recruited in this study, and 210 parturients were included in the clinical trial according to the inclusion criteria, with 105 patients in each group. In the follow-up stage, three subjects in the S group and five subjects in the L group were lost to follow-up after delivery. A total of 202 subjects were ultimately included in the statistical analysis. As shown in Figure 1.

There was no significant difference in age, height, weight, gestational times, gestational age, pre-operative hemoglobin, obstetric diseases, neonatal Apgar score, operation time, or intraoperative blood loss between the two groups, as shown in Table 1.

**TABLE 1:** Demographic data.

	Group S (*n* = 102)	Group L (*n* = 100)	*P* value
Age (years)	28.9 ± 3.9	29.6 ± 3.9	0.202
Height (cm)	160.7 ± 4.5	159.7 ± 3.8	0.084
Weight (kg)	69.4 ± 9.6	68.9 ± 8.8	0.727
Gravid status	2 (1∼7)	2 (1∼7)	0.583
Gestational age (weeks)	39.1 (37∼40.86)	39.0 (37.29∼41)	0.081
Pre-operative hemoglobin (g/L)	123.1 ± 10.9	123.0 ± 10.4	0.937
Comorbid obstetric diseases	38 (37.3%)	38 (38.0%)	0.913
**Apgar score**			
1 min	10 (10∼10)	10 (10∼10)	1.000
5 min	10 (10∼10)	10 (10∼10)	1.000
Duration of surgery (min)	35 (21∼92)	33 (22∼84)	0.176
Hemorrhage during operation (ml)	210 (200∼700)	200 (200∼1000)	0.05

Data are presented as numbers (percentages), medians (range) or means ± standard deviations; comorbidities included impaired glucose tolerance, gestational diabetes mellitus, gestational hypertension, and reduced free T3 and T4 during pregnancy.

**FIGURE 2:**
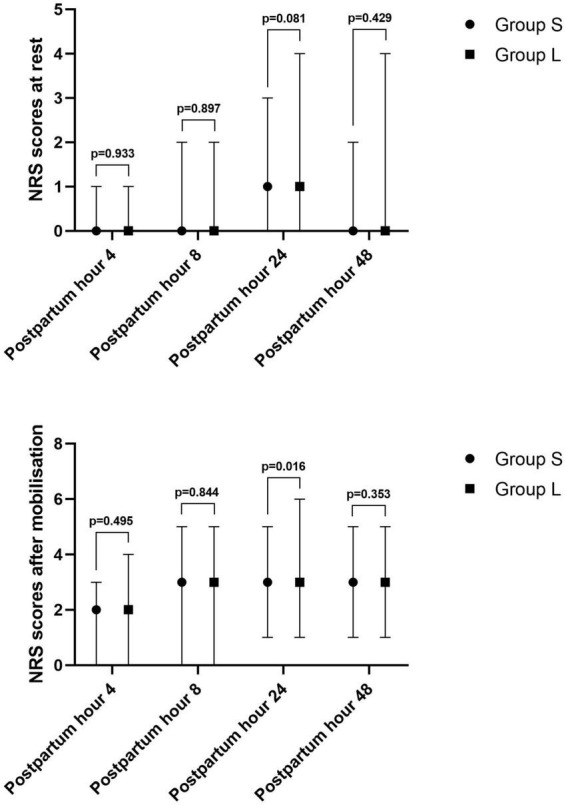
Post-operative pain scores (NRS) at rest and after mobilization in patients.

The NRS mobilization pain (cough pain) score in Group S was significantly lower than that in Group L at 24 h after the operation (*p* = 0.016), but there was no significant difference in NRS scores between the two groups at other times, as shown in Figure 2.

**FIGURE 3:**
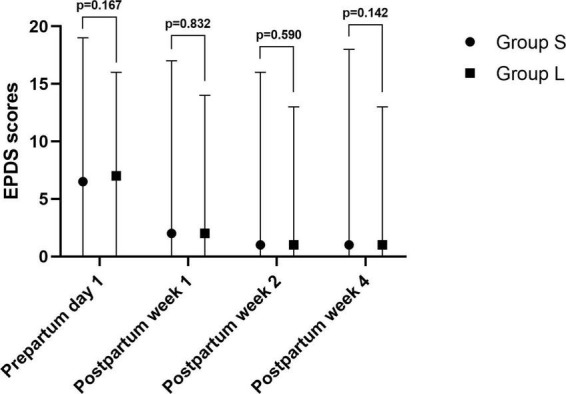
Edinburgh Post-partum Depression Scale (EPDS) scores in patients.

There was no significant difference in the incidence of depression between the two groups on the first day before delivery and the first, second, and fourth weeks after delivery, as shown in Table 2 and Figure 3.

**TABLE 2:** The prevalence of post-partum depression (PPD) between two groups.

	Group S (*n* = 102)	Group L (*n* = 100)	*P* value
Pre-partum day 1	26 (25.5%)	31 (31.0%)	0.384
Post-partum week 1	4 (3.9%)	2 (2.0%)	0.697
Post-partum week 2	2 (2.0%)	1 (1.0%)	1.000
Post-partum week 4	2 (2.0%)	1 (1.0%)	1.000

Data are presented as numbers (percentages).

The incidences of dizziness (*p* < 0.001) and hallucinations (*p* < 0.001) in Group S were higher than that in Group L at 5 min after esketamine administration. There was no significant difference in the incidences of nausea, headache, vomiting, diplopia, or blurred vision between the two groups. The incidences of dizziness (*p* < 0.001) and nausea (*p* = 0.011) 15 min after administration were higher in Group S than in Group L, but there was no significant difference in the incidences of hallucinations, headache, vomiting, diplopia, and blurred vision between the two groups. The incidence of dizziness (*p* < 0.001) when leaving the operating room in Group S was higher than that in Group L, while the incidences of hallucinations, headache, nausea, vomiting, diplopia, and blurred vision were not significantly different between the two groups, as shown in Table 3.

**TABLE 3:** Adverse effects in patients between two groups.

	Group S (*n* = 102)	Group L (*n* = 100)	*P* value
**Dizziness**			
5 min	50 (49.0%)	4 (4.0%)	<0.001
15 min	66 (64.7%)	2 (2.0%)	<0.001
When leaving the operating room	34 (33.3%)	0 (0.0%)	<0.001
**Hallucinations**			
5 min	28 (27.5%)	0 (0.0%)	<0.001
15 min	2 (2.0%)	0 (0.0%)	0.498
When leaving the operating room	0 (0.0%)	0 (0.0%)	1.000
**Nausea**			
5 min	2 (2.0%)	0 (0.0%)	0.498
15 min	18 (17.6%)	6 (6.0%)	0.011
When leaving the operating room	7 (6.9%)	3 (3.0%)	0.347
**Headache**			
5 min	0 (0.0%)	0 (0.0%)	1.000
15 min	1 (1.0%)	0 (0.0%)	1.000
When leaving the operating room	1 (1.0%)	0 (0.0%)	1.000
**Vomiting**			
5 min	0 (0.0%)	0 (0.0%)	1.000
15 min	0 (0.0%)	0 (0.0%)	1.000
When leaving the operating room	3 (2.9%)	4 (4.0%)	0.979
**Diplopia**			
5 min	0 (0.0%)	0 (0.0%)	1.000
15 min	2 (2.0%)	0 (0.0%)	0.498
When leaving the operating room	0 (0.0%)	0 (0.0%)	1.000
**Blurred vision**			
5 min	0 (0.0%)	0 (0.0%)	1.000
15 min	1 (1.0%)	0 (0.0%)	1.000
When leaving the operating room	1 (1.0%)	0 (0.0%)	1.000

Data are presented as numbers (percentages).

**FIGURE 4:**
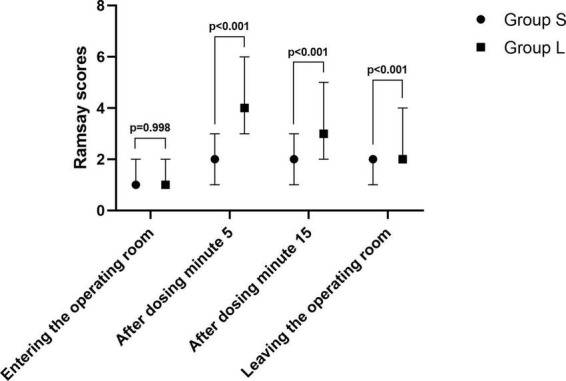
Ramsay scores at different time points.

The Ramsay scores in Group S were lower than in Group L at 5 min (*p* < 0.001), 15 min (*p* < 0.001) after dosing and at the time of leaving the operating room (*p* < 0.001). While the Ramsay score was not significantly different between the two groups when entering the operating room, as shown in Figure 4.

## Discussion

The results of this study showed that a single intravenous injection of 0.25 mg/kg esketamine during cesarean section (5 min. after neonatal delivery) did not reduce the incidence of PPD in the first, second, or fourth weeks after cesarean section, but significantly reduced the pain score for motor pain (cough pain) at 24 h after cesarean section. At the same time, compared with saline, the administration of ketamine at 5 and 15 min after treatment produced escitalopram ketamine-associated adverse reactions such as hallucinations, dizziness, and nausea, but eased quickly. A total of 33.3% of the mothers still felt dizzy at the time of leaving the operating room, but the degree was mild, the rest of the adverse reactions basically disappeared, and the time of leaving the operating room was not delayed. Interestingly, in this experiment, we also found that the Ramsay scores in Group S were lower than in Group L at 5 min, 15 min after dosing and at the time of leaving the operating room. Esketamine has a sedative effect, so it can significantly relieve the anxiety and tension of parturients during the perioperative period, and make patients calm, balanced, and more cooperative. In addition, we speculate that a low dose of esketamine will not affect the patient’s state of consciousness.

The DSM-5 (12) defines depression that occurs during pregnancy or 4 weeks after delivery as PPD. However, the International Statistical Classification of Diseases and Related Health Problems, 10th Edition (ICD-10) (13) defines PPD as depression occurring within 6 weeks after delivery. Therefore, in clinical practice and clinical studies, researchers’ definitions of the duration of PPD vary widely, ranging from 4 weeks to 12 months after delivery (7).

Although this study only observed the incidence of depression at 4 weeks post-partum, according to our clinical study, two sets of elective cesarean sections at 4 weeks found that the incidence of depression was 1∼2%. The reasons for the lower incidence of depression in this study may be: the observation time is not long, the needs of the newborn within 4 weeks are not high so that the mother can get enough rest, and the happiness of new mothers also helps to reduce the incidence of depression. However, despite this, depression can have serious effects on oneself and others, including low mood, anxiety and irritability, loss of interest, sleep and appetite disorders, psychomotor disorders, fatigue, guilt, and suicidal thoughts in 20% of perinatal MDD patients (14), and some women with PPD even have thoughts of harming their children.

Previous studies have found that a single intravenous dose of the antidepressant ketamine is 0.5 mg/kg, depressive symptoms are significantly relieved after 40–60 min, and the antidepressant effect can last for 5–8 days (15). Because the potency of esketamine in the human body is two times higher than that of ketamine (16) and the dose used to achieve the same anesthetic effect is half of that of ketamine, we used 0.25 mg/kg esketamine to study the occurrence of PPD. Singh (17) also found a strong antidepressant effect after 40 min of an intravenous infusion of 0.2 mg/kg esketamine in patients. Ma JH (18) also showed that prophylactic use of ketamine can reduce the incidence of PPD in women undergoing cesarean section. To date, there is no unified conclusion on the antidepressant mechanisms of ketamine and esketamine. Currently, it is generally accepted that (1) the inhibition of NMDA receptors located in γ-aminobutyric acid interneurons and NMDA receptor-dependent bursts of lateral cingulate neurons by inhibition of synaptic or selective GluN2B N-methyl-D-aspartate receptors activate AMPA (α-amino-3-hydroxy-5-methyl-4-isoxazole-propionate) receptors to exert antidepressant effects (10); (2) NMDA receptors are blocked and AMPA receptors are activated, and then neurotrophic effects are promoted, such as the release of brain-derived neurotrophic factor, eukaryotic elongation Factor 2, and other activated downstream nutrient signal cascades, resulting in dendritic growth and synaptic shape in the cerebral cortex (19, 20).

Our results found that a single injection of low-dose esketamine (0.25 mg/kg) and saline had no statistically significant difference in the incidence of PPD. A recent study by Wang QW (21) showed that intravenous injection of a small dose of esketamine after the delivery of the fetus can effectively reduce the risk of PPD, which is different from ours. We think it may be caused by the following reasons: (1) different screening criteria for PPD. Wang QW et al. defined a score of 10 and above as PPD. (2) the dosage of esketamine is different. We used 0.25 mg/kg, while Wang QW et al. used 0.5 mg/kg. We chose a more conservative but safe dose.

In addition, there are some differences between the results of this study and the Ma JH study, and the possible reasons are as follows: (1) different post-operative analgesic formulations. Ma JH et al. added 160 mg/10 ml ketamine to the post-operative analgesic pump in the ketamine group, while the control group did not receive ketamine. It is important to note that ketamine has been found to be neurotoxic to the neonatal brain (22), and its metabolites can be absorbed by newborns through breast milk. Therefore, the use of ketamine in analgesic pumps should be done with caution. Numerous animal studies in rodents have shown that ketamine induces neurodegeneration in the developing brain in a dose-dependent manner. Some post-natal preclinical studies have shown that ketamine induces brain apoptosis and brain damage in rodent infants (23, 24), but evidence on the neurological effects of esketamine on newborn brains is lacking. Therefore, the use of high-dose esketamine during the perioperative period to prevent PPD may not be appropriate for all women. In this study, esketamine was not added to post-operative analgesic pumps in either group, and esketamine is relatively new, although six studies have reported the safety of the intranasal use of esketamine (25–30). These studies showed that the adverse effects of esketamine were generally mild to moderate, usually occurred during and immediately after treatment, and resolved within the same day. Less than 5% of all patients treated with intranasal esketamine have serious adverse effects (26–30). Patients during the period of escitalopram ketamine treatment report adverse events, including hip fracture, significantly elevated blood pressure, ventricular premature beat, hypothermia, lacunar cerebral infarction, transient seizures, syncope, anxiety, agitation, aggression, calm, disorientation, and suicidal ideation, but whether these adverse events and escitalopram ketamine are directly related to it is still unclear. During 1 year of esketamine maintenance treatment (30), five patients (0.6% of the sample) had serious adverse effects related to esketamine, including anxiety, paranoia, delirium, suicidal ideation, and attempted suicide. However, according to the current literature, there are few relevant data on the safety of esketamine in post-operative analgesic pumps for patients in the ward, so it should be used with caution, at least in the present situation. (2) The observation time of PPD was different. There were significant differences in the onset time of PPD among different women, which may also lead to differences in the findings. (3) A therapeutic effect does not indicate a preventive effect. Esketamine has a definite effect on antidepressant treatment but not necessarily on the prevention of depression.

Ketamine reduces pain intensity by reducing NMDA receptor-mediated and opioid-induced hyperalgesia (31). In this study, there was no difference in the resting pain score between the two groups at 4, 8, 24, and 48 h after the operation, possibly due to the use of multimodal analgesia (epidural injection of hydromorphone and post-operative PCIA analgesia), but the score of motion pain (pain during cough) at 24 h after the operation was significantly lower in the experimental group than the control group. However, there was no significant difference in motor pain (cough pain) 48 h after the operation, which may be caused by the routine addition of non-steroidal anti-inflammatory drugs for analgesia by the responsible physician 24 h after the operation.

As mentioned above, pain (including pain in the entire perioperative period) is a risk factor for PPD. However, under the conditions of this study, there was no statistically significant difference in the incidence of PPD between the two groups of cesarean delivery women 4 weeks after delivery, which may be due to the low pain intensity in both groups caused by multimodal analgesia in this experiment. Even so, in this study, esketamine was found to relieve exercise pain 24 h after surgery and is still of important clinical significance; it can prevent low mood, anxiety and fatigue caused by pain, improve post-operative satisfaction, and promote maternal recovery and early lactation. Therefore, improved analgesia can improve the quality of life of post-partum women and provide better care for newborns while also having an important positive impact on public health.

Future research directions can focus more on the application of higher doses of esketamine for women who are likely to be depressed or unable to breastfeed to achieve targeted prevention of PPD.

## Limitations

Our study has several limitations. First, no esketamine-related adverse events were recorded after maternal exit from the operating room. The results of this study showed that the incidence of dizziness in Group S was higher than that in Group L at exit from the operating room, but no related adverse events were observed during post-operative follow-up. Second, there was no follow-up for pain after post-partum 48 h. Further studies are needed to investigate the effect of esketamine on the prevention of early pain and later chronic pain after cesarean section. Third, in this study, PPD was only followed up to 4 weeks after operation. Although the DSM-5 regulation was during pregnancy or within 4 weeks after delivery, there were also studies that observed puerperas up to 6 months or even 1 year after delivery. Therefore, the effect of a single intravenous injection of 0.25 mg/kg esketamine on PPD during elective cesarean section still needs longer follow-up observation.

Therefore, it is necessary to conduct further research to resolve these limitations.

## Data availability statement

The original contributions presented in this study are included in the article/supplementary material, further inquiries can be directed to the corresponding authors.

## Ethics statement

The studies involving human participants were reviewed and approved by Zhejiang Xiaoshan Hospital. The patients/participants provided their written informed consent to participate in this study.

## Author contributions

JSh and CS: drafting the manuscript. CS, JSh, JY, and JSu: study design. JSh and XL: critical revision of the manuscript. JSh, XL, and YW: statistical analysis. JY and JSu: obtained funding and study supervision. YW, SS, JY, and JSu: administrative, technical, or material support. All authors contributed to acquisition or analysis of data, contributed to the article, and approved the submitted version.
